# Freezing shortens the lifetime of DNA molecules under tension

**DOI:** 10.1007/s10867-017-9466-3

**Published:** 2017-09-08

**Authors:** Wei-Ju Chung, Yujia Cui, Chi-Shuo Chen, Wesley H. Wei, Rong-Shing Chang, Wun-Yi Shu, Ian C. Hsu

**Affiliations:** 1 Department of Biomedical Engineering and Environmental Sciences, National Tsing Hua University, 101, Section 2, Kuang-Fu Road, Hsinchu, 30013 Taiwan; 2 Department of Computer Science, Tufts University, 419 Boston Avenue, Medford, MA 02155 USA; 3 Institute of Statistics, National Tsing Hua University, 101, Section 2, Kuang-Fu road, Hsinchu, 30013 Taiwan

**Keywords:** DNA freezing effect, DNA lifetime, DNA integrity, Lambda DNA, Single molecule, DNA nanotechnology

## Abstract

DNA samples are commonly frozen for storage. However, freezing can compromise the integrity of DNA molecules. Considering the wide applications of DNA molecules in nanotechnology, changes to DNA integrity at the molecular level may cause undesirable outcomes. However, the effects of freezing on DNA integrity have not been fully explored. To investigate the impact of freezing on DNA integrity, samples of frozen and non-frozen bacteriophage lambda DNA were studied using optical tweezers. Tension (5–35 pN) was applied to DNA molecules to mimic mechanical interactions between DNA and other biomolecules. The integrity of the DNA molecules was evaluated by measuring the time taken for single DNA molecules to break under tension. Mean lifetimes were determined by maximum likelihood estimates and variances were obtained through bootstrapping simulations. Under 5 pN of force, the mean lifetime of frozen samples is 44.3 min with 95% confidence interval (CI) between 36.7 min and 53.6 min while the mean lifetime of non-frozen samples is 133.2 min (95% CI: 97.8–190.1 min). Under 15 pN of force, the mean lifetimes are 10.8 min (95% CI: 7.6–12.6 min) and 78.5 min (95% CI: 58.1–108.9 min). The lifetimes of frozen DNA molecules are significantly reduced, implying that freezing compromises DNA integrity. Moreover, we found that the reduced DNA structural integrity cannot be restored using regular ligation process. These results indicate that freezing can alter the structural integrity of the DNA molecules.

## Introduction

DNA molecules have been widely used in various fields such as drug delivery, therapeutic systems, molecular imaging, and biomolecule detection recently due to their high mechanical flexibility and unique assembly specificity [1, 2]. For example, aptamer, a short oligonucleotide, has been utilized to enhance the therapeutic efficiency of cancer treatments [3, 4]. During reagent preparation, to maintain DNA quality and reduce DNA damage induced by free radicals in aqueous environments, DNA samples are usually stored at −20 °C [5]. In ionizing radiation studies involving the use of long half-life isotopes, mixtures of DNA samples and isotopes have typically been frozen for up to 30 days at −70 °C for ^125^I compounds to accumulate sufficient isotope decays [6, 7]. Because of the common usage of freezing protocols, various engineering developments and scientific studies will benefit from understanding the effect of freezing DNA on its integrity.

The structural integrity of DNA plays a critical role in different nucleic acid nanotechnology applications. For example, site-specific DNA strands can be assembled into three-dimensional nanostructures, which can serve as the building blocks of advanced nanodevices [8]. The structural stability of nucleotide probes can also influence hybridization efficiency for molecular detection. Various factors, such as ionic strength, can influence the integrity of molecular structures, and several technologies have been developed to probe the integrity of polynucleotides [9, 10]. However, to probe the integrity of DNA at a single molecular level is still challenging for most current technologies.

In this study, we examined DNA integrity at the molecular level by using bacteriophage lambda DNA as a model molecule. Bacteriophage lambda DNA has been widely used in single-molecule experiments, with a few examples being the effect of force on intercalators [11–14], DNA binding of antibiotics [15], DNA-binding proteins [16–19], replication [20, 21], and structural changes under mechanical force [22, 23].

Double-strand breaks are often formed by closely spaced nicks. One method of detecting nicks, particularly if they are closely spaced, is to stretch the DNA molecules in a low ionic strength buffer. When DNA is stretched, sections containing clustered nicks denature locally and induce DSBs [24]. The integrity of DNA molecules is also influenced by the buffer being used. The ionic strength plays a major role in the stability of double-stranded DNA (dsDNA). Because DNA backbones are highly negatively charged in aqueous solutions, low salt concentrations result in a low degree of charge shielding of the backbones, thus making dsDNA less stable [25]. When dsDNA is subjected to external tension, low ionic strength buffers facilitate peeling and force induced melting near the nicks [26–28]. In addition, various surfactants, such as Tween 80, are widely used for macromolecule assembly and nanostructure fabrication [29–31]. The effect of such surfactants on DNA integrity is not clear.

Although plasmid DNA samples can easily be prepared in house to ensure superior quality control, lambda DNA samples are usually sourced commercially. Lambda DNA (48,502 bp), which is several times larger than typical plasmids, is prone to chemical and mechanical damage during purification and storage. Some samples are frozen after purification and then thawed in the lab before being used in assays; although this freeze/thaw cycle does not affect most experiments, it can result in nicks that can skew the results of some experiments, especially if the DNA molecules are to be subjected to tension [32, 33].

In this study, we investigated the effects of freezing on DNA integrity at the molecular level by measuring the sustaining times of DNA molecules through the use of dual-beam optical tweezers. We analyzed the difference between frozen and non-frozen samples, as well as the difference between two batches of non-frozen samples.

## Materials and methods

### Bacteriophage lambda DNA handling

Bacteriophage lambda DNA samples were purchased from New England Biolabs (NEB, N3011S). The standard catalog item was frozen at −20 °C. The non-frozen samples were special order and shipped at 4 °C. Upon arrival, the frozen sample was thawed and stored at 4 °C. All lambda DNA samples were stored in 100 mM Tris, 0.5 M NaCl, and 50 mM EDTA (pH 7.5) at 4 °C. Samples older than 1 year were discarded.

Overhangs of lambda DNA (14 μg) were annealed with complementary oligos with a single biotin at the 3′ end for 40 min in 50 μl of TE buffer (10 mM Tris, 1 mM EDTA, pH 8.0). Unbound oligos were washed twice with 50 mM Tris (pH 7.5) with Amicon Ultra centrifugal filter (Millipore, UFC510096) at 8100 g for 15 min at 4 °C. All samples were then treated by 400 units of T4 DNA ligase (NEB, M0202S) to repair nicks at 16 °C for 2.5 h in 40–50 μl of 1X T4 DNA ligase buffer (50 mM Tris-HCl, 10 mM MgCl_2_, 10 mM DTT, and 1 mM ATP, pH 7.5) in PCR tubes. T4 DNA ligase was inactivated with additional 6 μl of 0.5 M EDTA (pH 8.0) to bring the final concentration to 53–65 mM and washed with TE buffer twice with centrifugal filter at 8100 × *g* for 15 min at 4 °C. The final products were stored in 100 mM Tris, 0.5 M NaCl, and 10 mM EDTA (pH 7.5) at 4 °C and used within 2 weeks.

### Optical tweezers setup

**Fig. 1:**
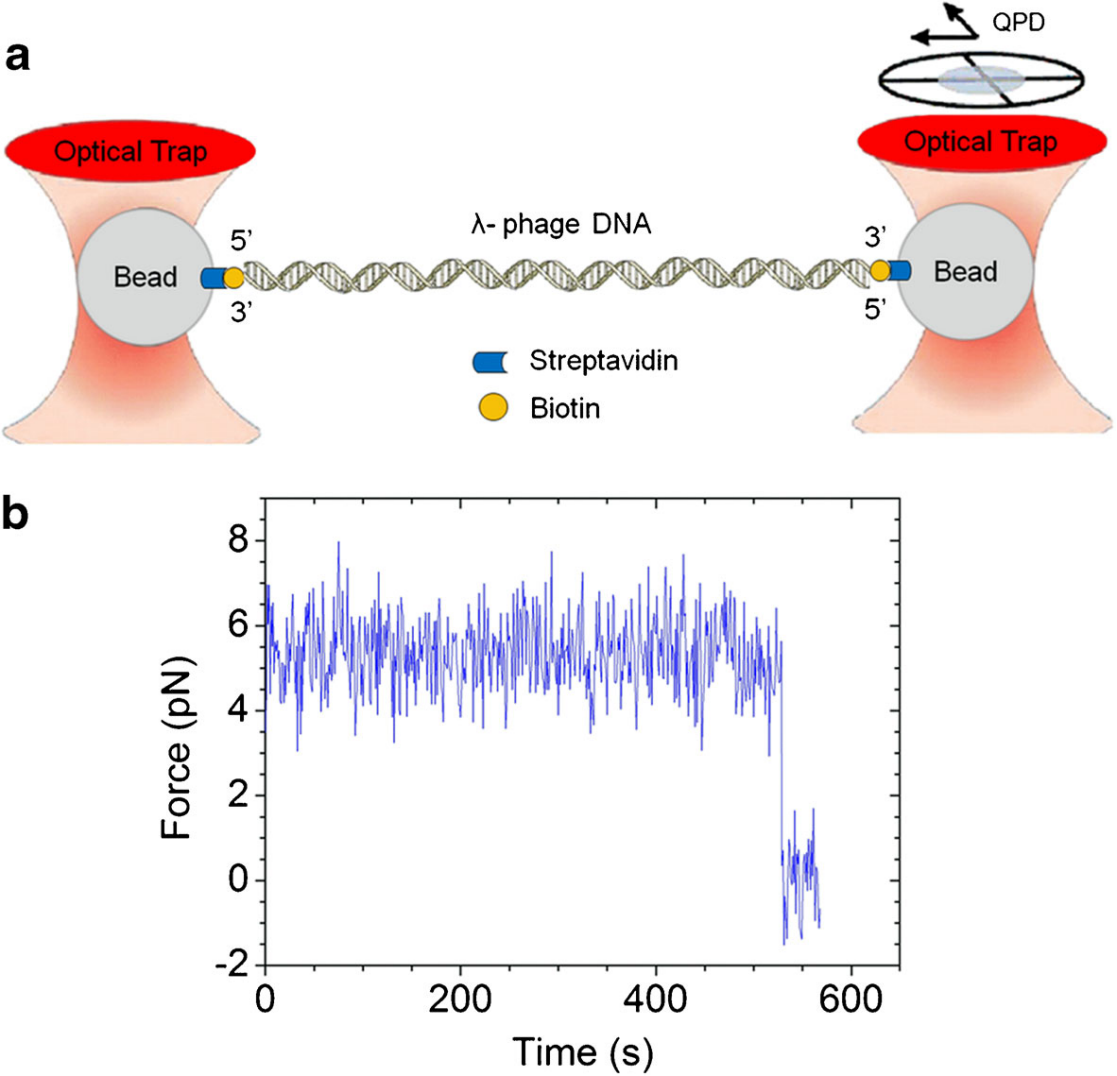
Measurement of sustaining time.** a** Schematic presentation of a single DNA molecule subjected to a constant force in dual-beam optical tweezers. The figure also illustrates the dumbbell structure of streptavidin-coated polystyrene beads with biotinylated lambda DNA between them. Both traps were formed by a neodymium-doped yttrium orthovanadate (Nd:YVO4) laser (1064 nm, Spectra-Physics). One beam was controlled by an acoustic-optical deflector (AOD, IntraAction Corp) to apply tension, and the other served as a fixed trap. The displacement of the bead in the fixed trap was detected by a QPD (model SPOT-9DMI, UDT), and used to calculate the force applied to the DNA molecule.** b** Sample trace of a single lambda DNA molecule subjected to a constant force. The force was 5.3 ± 0.9 pN, and the sustaining time was 528 s

The instrument setup was described by Yang et al. [34]. In short, the force-measuring dual-beam laser tweezers comprised one fixed trap and one movable trap, both formed by 1064-nm laser beams. A quadrant photodiode (QPD) was used to measure the signal from the position of the bead in the trap from an 830-nm defocused laser beam that was superimposed onto the fixed trap. Trap stiffness was calculated from the Brownian motion of the bead in the trap (Fig. 1a).

Labeled lambda DNA was incubated with streptavidin-coated beads (Spherotech, SVP-15-5) for approximately 1 h before being sealed in a liquid chamber containing TE buffer (10 mM Tris, 1 mM EDTA, 53 mM NaCl, and 0.3 mg/ml casein, pH 8.0) at 23 °C, with or without 4.6% Tween 80 (Sigma-Aldrich, P5188). Two beads were trapped, with a single DNA molecule suspended in between (Fig. 1a). The DNA molecule was then stretched to the desired tension by moving the movable trap. The force was deduced from the trap stiffness and the average position of the bead in the fixed trap.

Sustaining time was defined as the time required for a single DNA molecule to break under tension. In this type of measurement, a drop to zero in the force measurement indicated that the DNA molecule had broken (Fig. 1b). The cutoff time for measuring the DNA sustaining time was set to 60 min in this study.

### Data analysis

The probability that a DNA molecule survives time *t* was derived as follows [35, 36]:

1 $$ {P}_{model}(t)={e}^{-t/\tau } $$

where *τ* is the mean lifetime of DNA. This survival probability was estimated by:

2 $$ {P}_{data}(t)=\frac{n(t)}{N} $$

where *n(t)* is the number of DNA molecules not broken by time *t*, and *N* is the total number of molecules tested. The maximum likelihood estimate (MLE) of *τ* for the exponential probability distribution with censoring was given by:

3 $$ \widehat{\tau}=\frac{\sum_{i=1}^n{x}_i+{T}_w\left(N-n\right)}{n} $$

where *x*_*i*_ is the sustaining time of broken DNA, *T*_*w*_ is the period of measurement window (= 60 min in the study), *n* is the number of broken DNA molecules during the measurement period. The sampling distribution of $\hat{\tau}$ could be approximated by bootstrapping as follows: We generated 10,000 bootstrap samples, each of size *N*, from the exponential distribution with $\tau =\hat{\tau}$. For each of these samples, the maximum likelihood estimate, $\hat{\tau_b}$, of *τ* for the exponential distribution with censoring was calculated. The significance of differences among *τ*’s for various experimental conditions could be evaluated by their 95% confidence intervals (CI).

## Results

### Comparison of frozen and non-frozen samples

To evaluate the effects of the freezing protocol, frozen and non-frozen samples were compared in low-force experiments. The sustaining times of the frozen and non-frozen lambda DNA molecules were measured under tensile forces of 5 and 15 pN in low salt buffer. Mean DNA lifetime $\hat{\tau}$ was calculated according to Eq. (3). We evaluated the variance of lifetime under different exponential conditions using bootstrapping simulations. The results for frozen and non-frozen DNA molecules are shown in Table 1.

**Table 1:** MLE and variance of the mean lifetimes for frozen and non-frozen DNA samples under low force

Force	5 pN				15 pN			
DNA source	Mean lifetime *τ* (min)	95% CI (min)	% of sustaining time over 60 min	N*	Mean lifetime *τ* (min)	95% CI (min)	% of sustaining time over 60 min	N*
Frozen in TE	44.3	36.7–53.6	29%	143	10.8	7.6–12.6	0%	60
Non-frozen in TE	133.2	97.8–190.1	62%	100	78.5	58.1–108.9	47%	76
Non-frozen in TE with Tween 80	105.5	79.1–145.7	55%	100	75.1	58.0–98.6	42%	100

*Total number of DNA molecules studied

**Fig. 2:**
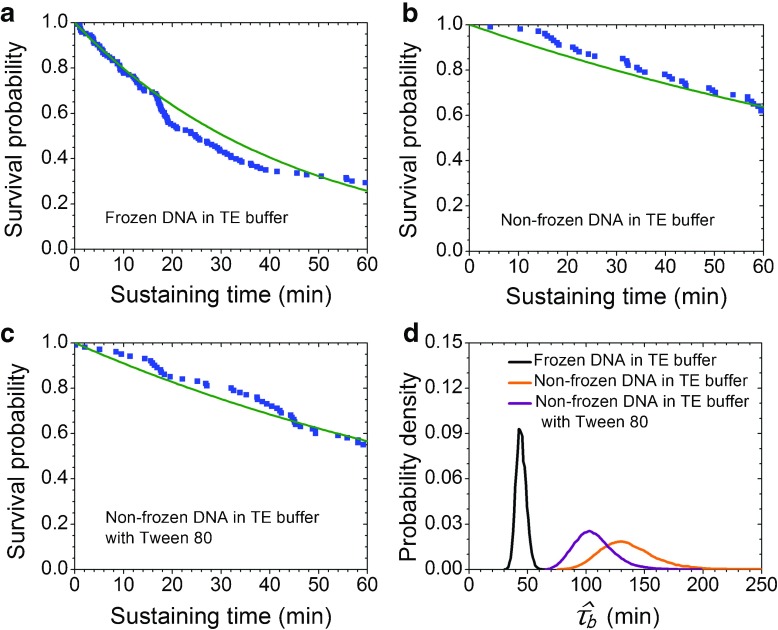
Survival probabilities of different DNA samples subjected to force of 5 pN. The* horizontal axis* for** a** to** c** is sustaining time of DNA and the* vertical axis* is survival probability. The* blue squares* represent the DNA sustaining time within the measurement window, and the* green lines* show the single exponential decay function with mean lifetime *τ* derived from maximum likelihood estimation.** a** Frozen DNA in TE buffer. There were 143 molecules examined and the MLE estimated lifetime is 44.3 min (95% CI: 36.7–53.6 min).** b** Non-frozen DNA in TE buffer. There were 100 molecules examined and the MLE estimated lifetime is 133.2 min (95% CI: 97.8–190.1 min).** c** Non-frozen DNA in TE buffer with 4.6% Tween 80. There were 100 molecules examined and the MLE estimated lifetime is 105.5 min (95% CI: 79.1–145.7 min).** d** The probability density, as approximated by bootstrapping, of the sampling distributions of $\hat{\tau}$ under various experimental conditions, where $\hat{\tau_b}$ is the maximum likelihood estimate of *τ* calculated from the bootstrap sample with censoring

Survival probability plots were generated by plotting survival probabilities versus time, see Fig. 2a-c. Survival probabilities from experiments are represented by blue squares. The green line is the exponential decay function with $\hat{\tau}$ as decay constant. For each experimental condition, the distributions of those 10,000 bootstrap estimates of mean lifetime were plotted. The results for 5 pN are shown in Fig. 2d. The distribution of frozen DNA samples is distinct from both non-frozen samples under the same tension. Although the mean of the two non-frozen samples are different, their 95% confidence intervals overlap. Our data indicated that there is a significant difference between frozen and non-frozen samples, and no significant difference between samples stored in TE buffer and TE with Tween 80. Bootstrap simulations for 15 pN show similar results. The 95% confidence intervals of sampling distribution from bootstrapping of the frozen sample separate completely from those of the non-frozen samples.

Regarding the data derived for the samples in TE buffer at a tensile force of 5 pN, the only difference between the samples was that the frozen samples were stored and shipped at −20 °C, whereas the non-frozen samples were maintained at 4 °C. The results of MLE lifetime show that frozen samples have a shorter lifetime (44.3 min, 95% CI: 36.7–53.6 min), then non-frozen samples in the same buffer (133.2 min, 95% CI: 97.8–190.1 min) (Table 1). Only 29% of the frozen DNA molecules lasted more than 60 min under the 5 pN force, whereas 62% of the non-frozen samples in TE buffer lasted more than 60 min under this force.

We studied the effects of surfactants on DNA integrity by using non-frozen lambda DNA with additional Tween 80 (4.6%) in the liquid chamber. Compared with a sample without Tween 80, the lifetime of the non-frozen samples was slightly shortened to around 105.5 min (95% CI: 79.1–145.7 min), and the number of molecules that survived past 60 min in the sample with Tween 80 dropped slightly from 62% to 55%. Despite the 21% reduction in the DNA lifetime in the presence of Tween 80, the shortened lifetime was still more than two times longer than that of the frozen samples in TE buffer.

To further investigate the mechanical strength of the DNA molecules, we increased the tension exerted on the molecules. We observed the differences in lifetime between the frozen and non-frozen samples under a force of 15 pN. All the frozen samples in TE buffer broke within 60 min and 47% of the non-frozen samples in TE buffer survived over 60 min. The addition of Tween 80 slightly reduced the amount of samples that survived past the 60 min mark to 42%. The lifetime of the frozen samples was 10.8 min (95% CI: 7.6–12.6 min), considerably shorter than that of the non-frozen samples (78.5 min, 95% CI: 58.1–108.9 min).

Increasing the mechanical force shortened the lifetimes of the molecules, an observation that is consistent with those reported in the literature [37, 38]. Because the non-frozen samples subjected to the 5 and 15 pN force experiments were derived from the same batch of lambda DNA, the differences in their lifetimes should be directly related to the different tensile forces applied. Moreover, this result implies that the freezing protocol made the DNA molecules more fragile under higher tension.

**Fig. 3:**
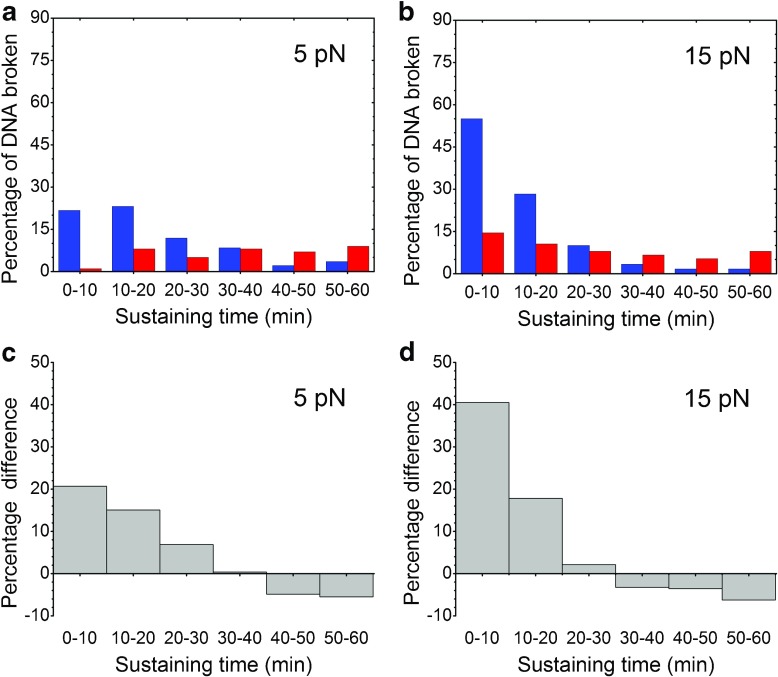
Sustaining times of frozen and non-frozen lambda DNA samples under constant tension. The* x*-axis represents the DNA sustaining time (min), and the* y*-axis represents the percentage of all DNA molecules tested in TE buffer.* Blue histograms* represent frozen DNA samples, and* red histograms* represent non-frozen samples.** a** Histogram for frozen samples subjected to a tensile force of 5.0 ± 0.7 pN and non-frozen samples subjected to a force of 5.3 ± 1.1 pN.** b** Histogram for frozen samples subjected to a force of 15.3 ± 1.5 pN and non-frozen samples to a force of 15.1 ± 0.7 pN.** c** Differences in the percentage of broken DNA between frozen and non-frozen samples at 5 pN.** d** Differences in the percentage of broken DNA between frozen and non-frozen samples at 15 pN

The histograms of the sustaining time are illustrated in Fig. 3a and b. All data within a histogram were derived from the same batch. Histograms of the frozen samples are presented in blue and those of the non-frozen samples are presented in red. For easy comparison, the numbers of DNA breakage events were replaced by the percentages of DNA molecules that were broken during the first 60 min of the experiments.

Figure 3a presents a comparison of the distributions of the sustaining times of the frozen and non-frozen DNA samples at 5 pN. From 0 to 30 min, more frozen DNA molecules were broken, compared with the non-frozen molecules. A lower percentage of frozen DNA molecules exhibited breakage between 40 and 60 min, presumably because most of the frozen molecules had already broken before this period. By contrast, the non-frozen samples exhibited a more even distribution across the 60-min observation, except for the first 10 min, during which only 1% broke. The differences in the sustaining time histograms between the two sample groups are shown in Fig. 3c. The greatest difference was observed within the first 10 min, decreasing as the sustaining time increases, and finally reaching a negative level after 40 min.

Similar differences were also observed between the frozen and non-frozen samples at 15 pN. Compared with the percentage observed at 5 pN, a greater percentage (55 vs. 22%) of the frozen DNA molecules broke within the first 10 min (Fig. 3b). After 20 min, most of the frozen molecules had already broken. For the non-frozen samples, more molecules were broken within 10 min compared with the results observed at 5 pN for samples from the same batch; however, approximately three-fourths of the molecules survived beyond 20 min. The differences between the two histograms are illustrated in Fig. 3d. Similar to the differences in Fig. 3c, the differences decreased as the sustaining time increased. After 30 min, most of the frozen molecules had already broken, thus resulting in fewer molecules to be observed.

The lifetimes of the frozen lambda molecules were observed to be significantly shorter than those of the non-frozen samples at 5 and 15 pN. Our data show that the freezing process, even without repeated freeze/thaw cycles, made a significant impact on DNA integrity when tested in a tensile force range common in biologically relevant processes.

### Comparison of non-frozen samples from different batches

**Fig. 4:**
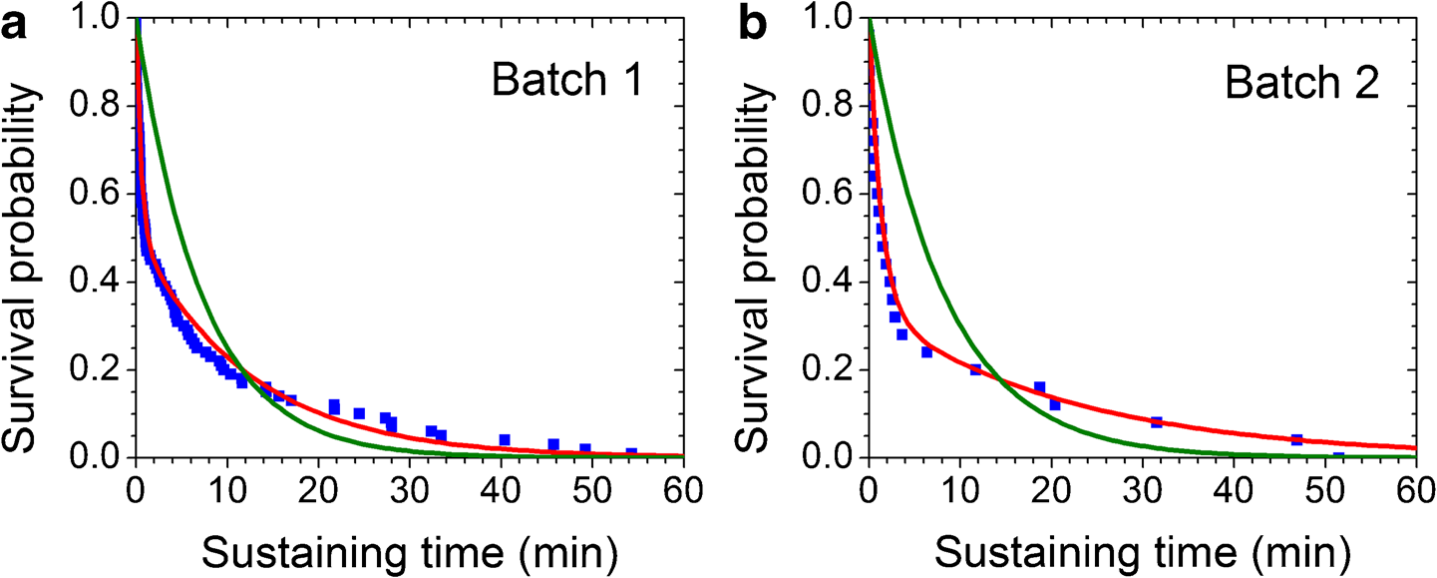
Survival probability of different DNA batches under 35 pN of force. The* horizontal* and* vertical axes* are sustaining time and survival probability of DNA. The* blue squares* represent DNA sustaining times observed in experiments. The* green lines* show the single exponential decay function with the MLE estimated lifetime *τ*; the* red lines* show the sum of two exponential fitting for data.** a** Batch 1 of non-frozen DNA (100 molecules) in TE buffer with 4.6% Tween 80. The MLE estimated lifetime was 7.2 min. The sum of two exponential decay fitting gave lifetimes of 0.53 ± 0.02 min and 12.5 ± 0.5 min.** b** Batch 2 of non-frozen DNA (25 molecules) in TE buffer with 4.6% Tween 80. The MLE estimated lifetime was 8.3 min. The sum of two exponential decay fitting gave lifetimes of 1.3 ± 0.1 min and 22.3 ± 4.0 min

The sustaining times of non-frozen DNA samples from two batches purchased from the same vendor were measured at 25 and 35 pN to ensure that most, if not all, molecules would break before the cutoff time (Table 2). All samples were processed in exactly the same manner from their arrival in the lab to their loading in liquid chambers; moreover, all buffers used were the same. A single exponential decay probability function with MLE lifetime do not fit data sets well, as shown by green lines in Fig. 4a and b. The red lines in Fig. 4a and b show that the sum of two exponential decay functions is a much better fit for the data, which implies that more than one mechanism may contribute to the observed DNA breakage. Because all DNA molecules tested broke within the measurement window of 60 min for both batches, it is not necessary to apply the MLE method. Sample lifetimes were calculated by fitting the survival probability plots to the sum of two exponential decay functions.

**Table 2:** Lifetimes of non-frozen DNA samples from different batches in TE buffer with Tween 80

Force	25 pN					35 pN				
Batch	Fitting ratio of population	Lifetime *τ*_*1*_ (min)^$^	Fitting ratio of population	Lifetime *τ*_*2*_ (min)^$^	N*	Fitting ratio of population	Lifetime *τ*_*1*_ (min)^$^	Fitting ratio of population	Lifetime *τ*_*2*_ (min)^$^	N*
1	0.26	1.1 ± 0.1	0.74	19.7 ± 0.7	100	0.49	0.53 ± 0.02	0.51	12.5 ± 0.5	100
2	0.22	5.3 ± 1.7	0.78	26.9 ± 2.0	45	0.66	1.3 ± 0.1	0.34	22.3 ± 4.0	25

*Total number of DNA molecules studied

^$^Lifetime ± standard deviation from sum of two exponential decay fitting

Most of the molecules from both batches that were tested at the relatively high tensile forces of 25 and 35 pN broke within 60 min. The samples subjected to the 25 and 35 pN forces exhibited two discrete populations, a shorter lifetime group and a longer lifetime group. The effects of the applied forces on both batches were also similar. Specifically, the lifetimes of the samples from both batches were shortened when the tensile force increased from 25 to 35 pN. At 25 pN, batch 1 had lifetimes of 1.1 ± 0.1 min and 19.7 ± 0.7 min, whereas batch 2 had lifetimes of 5.3 ± 1.7 min and 26.9 ± 2.0 min. The differences were within the same order of magnitude and show no significant difference. The same trend was also observed at 35 pN. Batch 1 had lifetimes of 0.53 ± 0.02 min and 12.5 ± 0.5 min, whereas batch 2 had lifetimes of 1.3 ± 0.1 min and 22.3 ± 4.0 min. Overall, under the same tension, DNA in batch 2 had a longer lifetime than did that in batch 1, indicating that it was of higher quality in terms of structural integrity.

For batch 1, at 25 pN, 26% of the tested molecules were in the shorter lifetime group; the remaining molecules were in the longer lifetime group. When the tensile force increased to 35 pN, the sizes of the two populations evened out. For batch 2, although two populations were also observed, the profile was different from that observed for batch 1 at both forces. At 25 pN, a lower percentage of the tested molecules belonged to the shorter lifetime group (22 vs. 26%). At 35 pN, more than half of the molecules belonged to the shorter lifetime group.

**Fig. 5:**
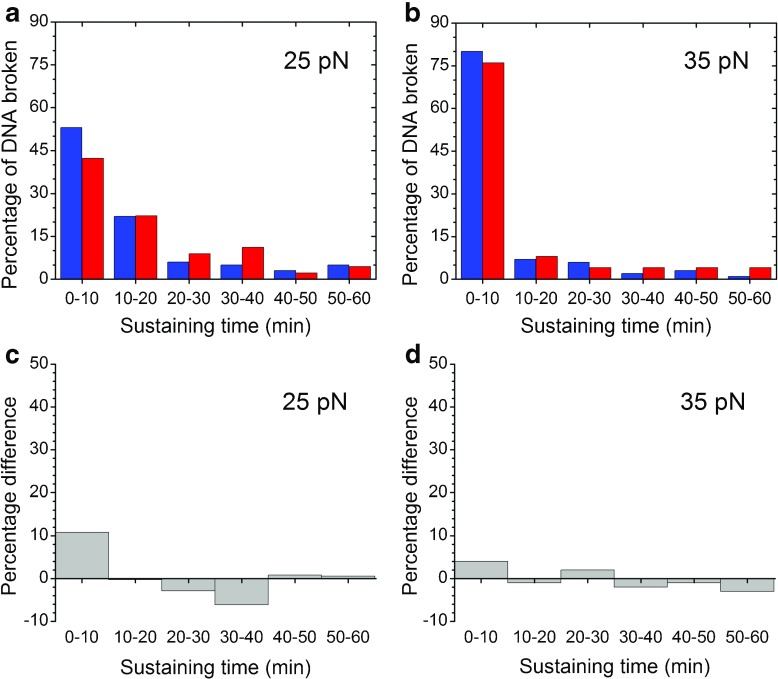
Sustaining times of non-frozen lambda DNA samples under 25 and 35 pN tension. The* x*-axis represents the DNA sustaining time (min), and the* y*-axis represents the percentage of all DNA molecules tested in TE buffer with 4.6% Tween 80.* Blue histograms* denote samples from batch 1, and* red histograms* denote samples from batch 2 in** a** and **b**.** a** Of 100 molecules tested from batch 1 at a tensile force of 24.6 ± 1.9 pN, 6% lasted over 60 min. Of 45 molecules tested from batch 2 at a tensile force of 24.9 ± 1.0 pN, 9% lasted over 60 min.** b** Of 100 molecules tested from batch 1 at a tensile force of 34.9 ± 1.8 pN, only one lasted over 60 min. All 25 molecules tested from batch 2 at a tensile force of 34.7 ± 1.7 pN were broken within 60 min.** c** Differences in the percentages of broken DNA between the two batches of non-frozen DNA samples at a tensile force of 25 pN.** d** Differences in the percentages of broken DNA between the two batches of non-frozen DNA at a tensile force of 35 pN

The histogram of the sustaining time shows the distributions of the populations within each sample. The histograms of the sustaining times of non-frozen lambda DNA molecules from the two batches are illustrated in Fig. 5a and b. All data within a histogram were derived from a single batch. The histograms of the samples from batch 1 are presented in blue and those of the samples from batch 2 are presented in red. For easy comparison, the numbers of DNA breakages were replaced by the percentages of DNA molecules that were broken during the first 60 min of the experiments.

Figure 5a presents a comparison of the distributions of the sustaining times of the non-frozen DNA samples at 25 pN, and Fig. 5c illustrates the difference between the two histograms. From 0 to 10 min, more DNA molecules broke in batch 1 than in batch 2. From 10 to 40 min, a lower percentage of DNA molecules from batch 1 broke, presumably because most of the molecules that had been frozen had already broken within 40 min. From 40 to 60 min, the number of molecules observable from both samples was extremely low; thus, the fluctuation may not bear any statistical significance. The histograms of the two batches at 35 pN are shown in Fig. 5b. The difference in the sustaining time histograms between the two sample groups at 35 pN is presented in Fig. 5d. The greatest difference was observed within the first 10 min. Beyond that time, the size of the sample was too small for us to draw clear conclusions.

## Discussion and conclusions

The considerable difference in lifetime between the frozen and non-frozen DNA samples at a low force could have a few underlying causes. We speculate that the most likely cause is the higher number of nicks in the frozen sample, which consequently makes it more likely to have a pair of closely spaced nicks on opposite strands. In low ionic strength buffer, a pulling force is conducive to breathing/peeling in dsDNA [39, 40] and accelerates the breaking process near closely spaced nicks. Our data indicate that the frozen DNA specimens had lower mechanical strength levels, which is consistent with previous findings for DNA at low temperatures [10].

DNA ligase is frequently used in laboratories to facilitate the formation of phosphodiester bonds of adjacent DNA bases, and ligation is expected to enhance DNA integrity. In our experiments, T4 DNA ligase is applied to repair the nicks of both the frozen and non-frozen samples as part of the biotinylation procedure, and this ligation procedure is expected to repair the nicks along the DNA structure.

The ligation condition used in this study, 16 °C for 2.5 h, is shorter than the recommended ligation time in the manufacturer’s protocol, and a more optimized ligation procedure may produce frozen samples with longer lifetimes. On the other hand, we cannot ignore the possibility that ligation procedures may not be able to fully restore the damages caused by freezing DNA samples. Therefore, if intended experiments rely on DNA integrity, avoiding samples that have been frozen is worthwhile.

Non-frozen samples offer higher DNA integrity than frozen samples do, but long DNA molecules from different batches still show different survivorship profiles under tension. In contrast to lower tensile force (5–15 pN), two discrete populations with different decay lifetimes were observed under higher tensile force (25–35 pN). These results suggest that more than one single factor may contribute to the DSBs, and we speculate that various DSBs mechanisms, such as bubble migration and strand peeling [28, 41], could contribute to the observed DSBs in our experiments. It is also possible that the rupture of single biotin-streptavidin binding between DNA and beads [42] contributes to the additional population as well. More experiments are needed to further explore the potential mechanisms of the DNA breakage under higher tensile force. Due to batch-to-batch differences, care should be taken to perform all experiments from one batch.

We used dual-beam optical tweezers to evaluate the integrity of DNA molecules by stretching single molecules in low ionic strength buffers. Our results demonstrate that common freezing protocols can reduce DNA integrity at the molecular level. When a moderate tensile force (< 20 pN) was applied to mimic the mechanical interactions between enzymes and DNA molecules, the lifetimes of frozen DNA molecules decreased dramatically. Considering the increasing applications of DNA molecules in numerous fields, our findings are expected to aid developments in sample preparation and storage procedures in DNA nanotechnology.
